# Reply to Letter to the Editor in reference to “The transotic approach for vestibular schwannoma: indications and results”

**DOI:** 10.1007/s00405-017-4711-8

**Published:** 2017-08-21

**Authors:** Yin Xia

**Affiliations:** Department of Otorhinolaryngology, Beijing Tiantan Hospital, Capital Medical University, Beijing, 100050 China

**Keywords:** Transotic approach, Vestibular schwannoma, Cerebellopontine angle, Cerebrospinal fluid leak

## Abstract

This communication is the response to letter to the editor in reference to “The transotic approach for vestibular schwannoma: indications and results”.

## Introduction

Thank you for your comments. However, our study was intended to show why we prefer to use the transotic approach (TO) rather than the translabyrinthine approach (TL) as described by House or one of his modifications. As you correctly point out, the advantage of the TO approach is the better exposure to the anterior cerebellopontine angle (CPA) permitting preservation of the Facial Nerve and radical tumor removal without or with minimal brain retraction [1, 2]. This advantage requires the sacrifice of the otic capsule anteriorly to the skeletonized intrapetrous facial nerve [3–5]. The TL approach with all his modifications does not allow the same exposure because of the preservation of the middle ear cavity particularly when MRI or HRCT scans show a limited access to the CPA (such as reduced pneumatization, low–middle cranial fossa, anterior sigmoid sinus, and/or high jugular bulb).

The recent modifications in the technique of the TL approach have learned from the Subtotal Petrosectomy introduced 1967 by Fisch that the elimination of the pneumatic spaces of the temporal bone is necessary to prevent postoperative cerebrospinal fluid leak complications [1]. However, the preservation of the middle ear cavity prevents the complete elimination of the pneumatic temporal bone spaces and limits the anterior access to the CPA in contracted mastoid.

This is why the references noted in the publication may be old but not necessarily outdated.

The tumor sizes of the vestibular schwannomas mentioned in the paper were measured on MRI images along the long axis of the tumor from the CPA to the fundus of the IAC. There are different tumor-grading systems according to tumor size now in the world. In addition, the tumor size is only a factor in our paper to decide whether the hearing would be sacrificed. How to choose different approaches according to tumor size and comparing with their effects were not the aims of our study.

The decision to sacrifice the hearing was taken in accordance with the patients when the hearing at the tumor side was lost or the hearing loss was more than 50 dB and the speech discrimination score was less than 50% taking also in account the better chance of total tumor removal and preservation of the Facial Nerve.

To decrease the incidence of complication, TL approach as described by House has been modified over the years. Prof. Sanna and his cooperators are experienced in treating vestibular schwannoma and they used the enlarged TL approach, which may reduce the incidence of CSF leak.

In conclusion, we think that our results, in spite of limitations in the study design, show that the transotic approach is not a modified translabyrinthine approach and provides convincing results in relation with total tumor removal, preservation of facial nerve function, avoidance of CSF leaks, and severe postoperative complications.

## References

[1] Fisch U. Subtotal petrosectomy with permanent anterior dislocation of the facial nerve and obliteration of the cavity. In: Naumann HH (eds) Head and neck surgery, vol III 1976 and 1982. G. Thieme Med Publ, Stuttgart, pp 544–551

[2] Jenkins HA, Fisch U (1980). The transotic approach to resection of difficult acoustic tumors of the cerebellopontine angle. Am J Otol.

[3] Bruce BJ, Fisch U (1983). Modified transotic approach to the cerebellopontile angle. Arch Otolaryngol.

[4] Fisch U, Mattox D (1988). Microsurgery of the skull base. Chapter 2 transotic approach.

[5] Fisch U, Joseph C, Brackmann DE, Shelton C, Arriaga MA (2010). Transotic approach. Otologic surgery. Chapter 51 transotic approach.

